# Resource demands in telco data centers

**DOI:** 10.1038/s41597-024-03493-9

**Published:** 2024-06-21

**Authors:** Dimitra Paranou, Angelos Pentelas, George Doukas, Konstantinos Chondralis, Dimitris Katsiros, Evangelos Angelou, Nikos Anastopoulos, Giorgos Giannopoulos, George Papastefanatos

**Affiliations:** 1 Athena Research Center, Information Management Systems Institute, Athens, Greece; 2 Intracom SA Telecom Solutions, Athens, Greece

**Keywords:** Business, Technology

## Abstract

Telecommunication (telco) cloud services have emerged as crucial components in the modern digital landscape, offering extensive capabilities for data management, connectivity, and service provision. However, research on telco clouds lacks comprehensive data on the characteristics of production workloads, which is fundamental for designing effective resource management systems, such as workload schedulers and power management mechanisms. To this end, this paper addresses a substantial gap in telco cloud research by creating a comprehensive dataset that encapsulates crucial information regarding the pattern demands of applications within telco data centers. In addition, the proposed dataset contributes to the field by enabling strategic network configuration, optimizing data center sizing, facilitating proactive decision-making for data center operations, but its applicability extends beyond these cases. These examples illustrate the practical value of the dataset in enhancing efficiency, reducing operational costs, and ensuring optimal performance within telecommunication data centers.

## Background & Summary

The rapid expansion of telecommunication (telco) data centers (DCs) is intrinsically tied to the evolution of Network Function Virtualization (NFV)^1^. NFV constitutes a cloud computing paradigm enabling the creation of network functions in the form of virtual machines and containers (i.e., virtual network functions - VNFs), and it has evolved within a broader technological ecosystem that includes the virtualization of the mobile core^2^. As such, the scope of NFV has expanded beyond traditional network functions (e.g., firewalls, and load balancers) and now includes additional applications many of which are data-intensive^3^. For these applications, it would be more efficient to run some VNFs at the edge of the network, i.e., closer to data sources. Through this lens, telco networks undergo a drastic transformation process including the introduction of telco edge clouds which, in addition, are meant to host cloud-native applications. Telco edge DCs bring network and processing capabilities closer to end users than typical centralized DCs, which may cause transmission delays owing to large distances. This makes it possible to respond in almost real-time, which is essential for applications like Internet of Things and 5G. In addition to optimizing latency, NFV and edge computing enhance the general scalability and agility of telecommunication networks. In other words, programs may now adapt their capacity on the fly to meet changing demand. Established scaling strategies provided by Virtual Infrastructure Managers (VIMs), like Kubernetes^4^, enable this flexibility.

In the competitive landscape of telco services, communication service providers (CSPs) face pressure to deliver compelling features and services while effectively managing DC energy and costs^5^. CSPs accommodate a diverse array of workloads originating from both external clients and internal services, all sharing a common infrastructure. Maintaining optimal performance, availability, and reliability under these conditions necessitates sophisticated yet practical and scalable resource management strategies^6,7^.

However, existing research in resource management within telco DCs lacks a comprehensive understanding of the fundamental characteristics of these workloads^3,8^ and is mainly focused on general DC network workloads^9,10^ or on static traffic scenarios^11^. Key aspects such as the lifespan (time from creation to termination) or consumption patterns of resources by production applications remain largely unexplored in prior studies. The absence of detailed data and analyses regarding workload patterns and resource utilization hampers the development of effective resource management systems tailored to the specific demands of telco DCs.

The primary objective of the proposed dataset is to replicate real-life demand patterns observed in operational telco DCs. This goal is driven by the inherent challenges of private telco datasets, making open-sourcing or sharing them under Non-disclosure agreements (NDAs) difficult. To illustrate the importance of this dataset and its intended users, consider situations where telecommunication providers and data center operators could gain advantages from a comprehensive collection of simulated yet realistic telco workloads. For instance, telecom service providers could utilize this dataset to enhance resource management efficiency by optimizing server allocation, predicting future demands, and minimizing operational costs. DC operators, on the other hand, can leverage the dataset to fine-tune their infrastructure, ensuring seamless operations while maintaining energy efficiency. The dataset is produced by deriving input traffic load patterns from real-life data obtained from an operational subscriber-driven telco service and then simulating these workloads in an AWS environment with Kubernetes deployments. This approach ensures that the dataset not only captures the intricacies of actual telco workloads but also offers practical insights for improving resource management strategies.

## Methods

### Workload Assumptions

In telco edge DCs workloads often exhibit periodic patterns, mainly stemming from the subscriber-driven nature of telco services. Cloud-native applications in such environments demonstrate the ability to dynamically scale their resources in response to the fluctuating load. The two prevailing scaling practices in modern virtualized infrastructures are horizontal and vertical scaling. Horizontal scaling enables the creation of new and the termination of existing application instances, whereas vertical scaling enables the dynamic allocation of resources to existing instances. Throughout this work, the horizontal scaling paradigm was adopted, since it is admittedly more common in Kubernetes environments.

**Fig. 1:**
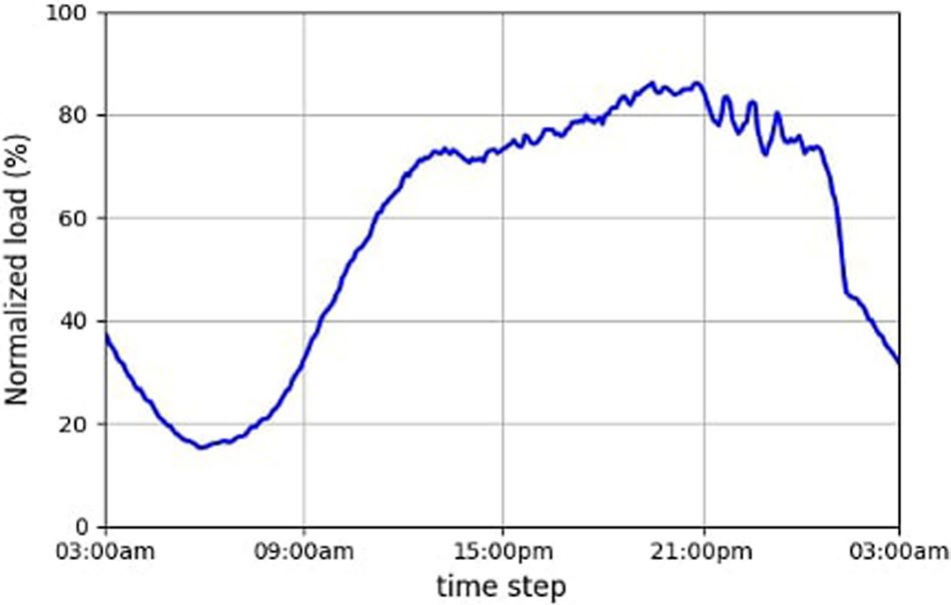
Daily load of a subscriber-driven service running in production (normalized values).

Initially, access was granted to traffic load metrics from a subscriber-driven service deployed within the production environment of a network operator. This service is a VNF operating at the data plane of the 5G mobile core. Notably, load metrics exhibit substantial fluctuations, with lower loads observed during early morning hours and significant peaks during late evening hours, as shown in Fig. 1.

Within this context, a service deployment entails multiple service instances, each capable of handling a specific fraction of the overall load. For example, we can assume that each instance is intended to manage up to 10% of the total load. Further, the service orchestrator often performs automated tasks related to load threshold setting and reactive scaling to carry out the practice of horizontal scaling, which is defined as raising or lowering the number of application instances in response to fluctuating load. This implies homogeneity among service instances, ensuring a predictable performance for each instance. Consequently, all instances have fixed resource requirements, typically encompassing CPU cores, memory, and DPDK-enabled interfaces. Since such services make up the majority of telco infrastructures, similar patterns are used to simulate several subscriber-driven services. These might include components of the 4G Evolved Packet Core, various elements within the 5G mobile core, and additional edge services pertaining to Industry 4.0. Crucially, the resource consumption pattern of any application is dependent on a pattern of instances, that are in turn driven by a pattern of total application load.

### Load Patterns

The set of applications *A* is considered with resource requirements *R* = {*CPUcores*, *memory*, *GPU*} and are used to produce the input traffic workloads in the telco DC. With the assumption that the load is not totally random, load patterns can be decomposed into three categories: diurnal, staggered, and fixed, each carrying a distinct variance level.

- **Diurnal Demand Patterns**. These patterns are defined by their adherence to a 24-hour cycle, reflecting the daily levels of user activity. They are marked by significant variability. For example, this pattern can be seen in deployments implementing VoIP and Mobile Core services, which typically experience lower activity during late night and early morning hours.
- **Staggered Demand Patterns**. Staggered demand patterns are characterized by their inverse relation to the, arguably more common in telco DCs, diurnal patterns. Workloads dictated by staggered patterns are strategically scheduled to complement peak traffic times, often during periods of low demand. These deployments are scheduled diametrically opposed to typical diurnal workloads, without impacting ongoing high-priority services. An example of a deployment that follows a staggered demand pattern is a daily backup process, which is typically run during off-peak hours to ensure resource availability and minimize service disruption.
- **Fixed Demand Patterns**. In contrast to the variable nature of diurnal and staggered patterns, fixed demand patterns exhibit a consistent resource demand level over time, as it is assumed that they are not influenced by exogenous variables such as the user traffic. Fixed demand patterns cater to critical services that require continuous operation, such as database clusters that cannot be shut down. Additionally, fixed demand patterns may also be found in conservative and inflexible deployments, which are meant to handle worst-case loads. The predictability of fixed patterns translates to a stable base load for DC operations.

### Workload Deployment

As evidenced by recent research^12–14^, telco operators embrace the agility, scalability, and container orchestration capabilities offered by Kubernetes. The paradigm of cloud-native telco infrastructures was adopted, and a dataset tailored to Kubernetes-based VIMs was devised. To this end, the term *node* was used to refer to Kubernetes compute nodes, and the term *deployment* was used to refer to applications. Compute resources are consumed by *replica pods*, since each deployment is realized via a set of replica pods whose size varies across time in response to volatile loads, as per the horizontal scaling practice in Kubernetes. Additionally, a set of nodes that are logically organized into a single group is referred to as *node cluster* or simply *cluster*.

Herein, the deployments (applications) that were utilized to create synthetic resource demand are discussed. Importantly, it is punctuated that the number of replica pods of each deployment varies based on loads observed in real-life telco deployments.

Six distinct Kubernetes deployments are implemented, each defined by specific resource demands per replica pod, a maximum pod count, and a load pattern type, detailed in Table 1. For instance, the *base-diurnal* deployment is realized via a set of replica pods each requesting 1 CPU thread and 1GB of memory. The load pattern of the *base-diurnal* is of type *diurnal*, implying that the load of this deployment is low during early morning hours, it progressively increases and peaks at late evening hours, then drops again and repeats this cycle throughout the experiment. As the load *l*_*t*_(*a*) of a deployment *a* varies within its normalized values range $\left(0,1\right]$, the number of the corresponding replica pods is computed via:

$${I}_{t}(a)=\lceil {l}_{t}(a)\cdot \,\mathrm{Max\ Pods}\rceil $$

This assumes that each replica pod can manage a portion of the overall deployment load, with the spawning and termination of replica pods following the practice of horizontal scaling. For example, in Scenario A, if *l*_*t*_(CPU-intensive) = 0.6, then the *CPU-intensive* requires ⌈0.6 ⋅ 25⌉ = 15 replica pods to serve its load, whereas in Scenario B, the corresponding number of the same deployment for the same load level is ⌈0.6 ⋅ 5⌉ = 3.

**Table 1:** Deployment demands (per replica pod).

Scenario	Deployment	CPU threads	Memory (GB)	GPU	Max Pods	Load pattern
Scenario A (dynamic)	base-diurnal	1	1	0	100	diurnal
	CPU-intensive	8	4	0	25	diurnal
	Memory-intensive	4	8	0	30	diurnal
	GPU-intensive	2	8	1	2	diurnal
	base-staggered	1	1	0	30	staggered
	base-fixed	1	1	0	70	fixed
Scenario B (static)	base-diurnal	1	1	0	10	diurnal
	CPU-intensive	8	4	0	5	diurnal
	Memory-intensive	4	8	0	5	diurnal
	GPU-intensive	2	8	1	2	diurnal
	base-staggered	1	1	0	30	staggered
	base-fixed	1	1	0	420	fixed
Scenario C (balanced)	base-diurnal	1	1	0	70	diurnal
	CPU-intensive	8	4	0	8	diurnal
	Memory-intensive	4	8	0	18	diurnal
	GPU-intensive	2	8	1	2	diurnal
	base-staggered	1	1	0	30	staggered
	base-fixed	1	1	0	284	fixed

Staggered patterns are effectively inverted diurnal patterns, whereas fixed patterns, as their name implies, do not have any load volatility and they are always realized via Max Pods replica pods. By using different distributions of Max Pods over the various deployments, different evaluation scenarios can be designed, since these affect the overall pattern of aggregated resource demands. For example, in a dynamic scenario (i.e., Scenario A), pods of diurnal deployments yield the biggest fraction of resource demand compared to staggered and fixed, and high variations in overall resource demands are anticipated. Conversely, in a static case (i.e., Scenario B), most demands are attributed to pods of the *base-fixed* deployment, hence small variations in overall resource demands are expected.

For the sake of completeness, note that stress-ng was utilized to create replica pods in Kubernetes. The resource demands of each replica pod are configured based on resource requests and limits specified in the corresponding Kubernetes deployment configuration.

## Data Records

**Fig. 2:**
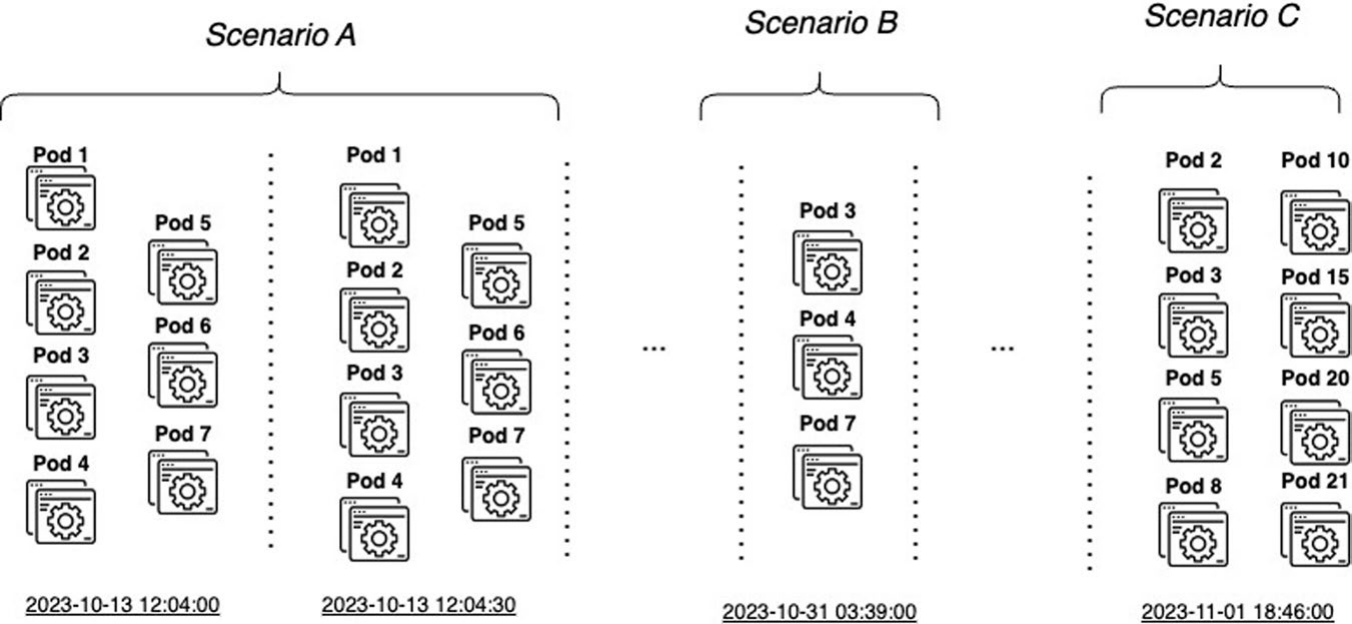
Visualized example of pod request file. In every timestamp, there are several pods with specific demands and every timestamp is grouped in a scenario case. Every scenario presents a varied workload, showcasing the nature of pod requests in telco data centers.

A dataset of pod resource demands for a period of approximately 20 days (i.e., from 2023-10-13 12:04:00 to 2023-11-02 08:46:00) is available at our Zenodo repository^15^. Specifically, data files containing detailed information regarding pod resource requests on a daily basis were exported. Data points are collected at a 30-second granularity. The files are collated, organized, and categorized based on scenarios and pod classifications. The pod file consists of seven columns, presenting details about CPU, memory, and GPU demands of individual pods at a given timestamp, along with the respective serving node that is mentioned in the *Node* column. The *Scenario* column distinguishes rows accordingly based on the workload scenario. The study considers 3 scenarios; Scenario A, where resource demands are highly dynamic, Scenario B, where resource demands are static, and Scenario C, the balanced scenario, where demands are moderately dynamic. In the corresponding data repository, the Pods request dataset is stored in a zip .csv file titled *pods_request_workloads*, and the rows are sorted based on the timestamp column. There are no missing values, meaning that every pod (UID) corresponds to a Node; one Node can serve multiple pods, but one pod can be served by only one Node at each timestep. Figure 2 represents a visualized example of the data, where for every timestamp multiple pods have specific demands, and Table 2 illustrates a fraction of the dataset. Every pod has constant resource demands which do not change across its lifespan.

**Table 2:** Sample of file *pods_request_workloads.csv* that stores information about pods requests sorted by the time.

Timestamp	UID	Node	CPU (cores)	Memory (Mb)	GPU (unit)	Scenario
2023-10-13 12:04:00	0144ce9b-3ac2-485a-b77f-f68ce5f15278	yUgR3woFgQ	0.1	0	0	A
2023-10-13 12:04:00	0181bc61-f566-4bde-a138-a3286faaf3e3	w7e7FPgTUy	1.0	1024	0	A
2023-10-13 12:04:00	02f4d0dd-a307-4917-acee-66d52e1f420a	eYSIRVqBog	2.0	8192	1	A
2023-10-13 12:04:30	0144ce9b-3ac2-485a-b77f-f68ce5f15278	yUgR3woFgQ	0.1	0	0	A
2023-10-13 12:04:30	0181bc61-f566-4bde-a138-a3286faaf3e3	w7e7FPgTUy	1.0	1024	0	A

## Technical Validation

The dataset is designed to meet specific requirements in the telco domain, and therefore the hosting infrastructure, the number and types of servers, and the workload characteristics are carefully chosen to be typical for such a use case. Amazon Web Services (AWS) is used to emulate a virtualized DC, and the relevant data is collected and stored using Prometheus (i.e., a time-series database) leveraging the OpenTelemetry API. The deployments of Scenarios mentioned in Table 1 are executed in a Kubernetes cluster consisting of 34 AWS EC2 nodes. Each deployment comes with its own overall traffic pattern (i.e., diurnal traces resemble the real-life traffic trace from the operational telco service, staggered traces are inverse diurnal, whereas fixed traces are static). For the first few days all nodes are active despite the highly volatile resource demand. During this period, replica pods of various Kubernetes deployments are spawned and terminated through horizontal scaling, and we resort to the default Kubernetes Scheduler to assign pods to nodes. Throughout this initial phase of the experiment, the resource demands of pods are recorded and stored into Prometheus. As such, it becomes apparent that the proposed dataset originates from actual workloads deployed on an operational infrastructure. This holds true both for the initial phase of experimentation and for the subsequent optimization phases. In the following subsection, node and application instances (Pods) characteristics are discussed in-depth.

### Infrastructure Setup

**Fig. 3:**
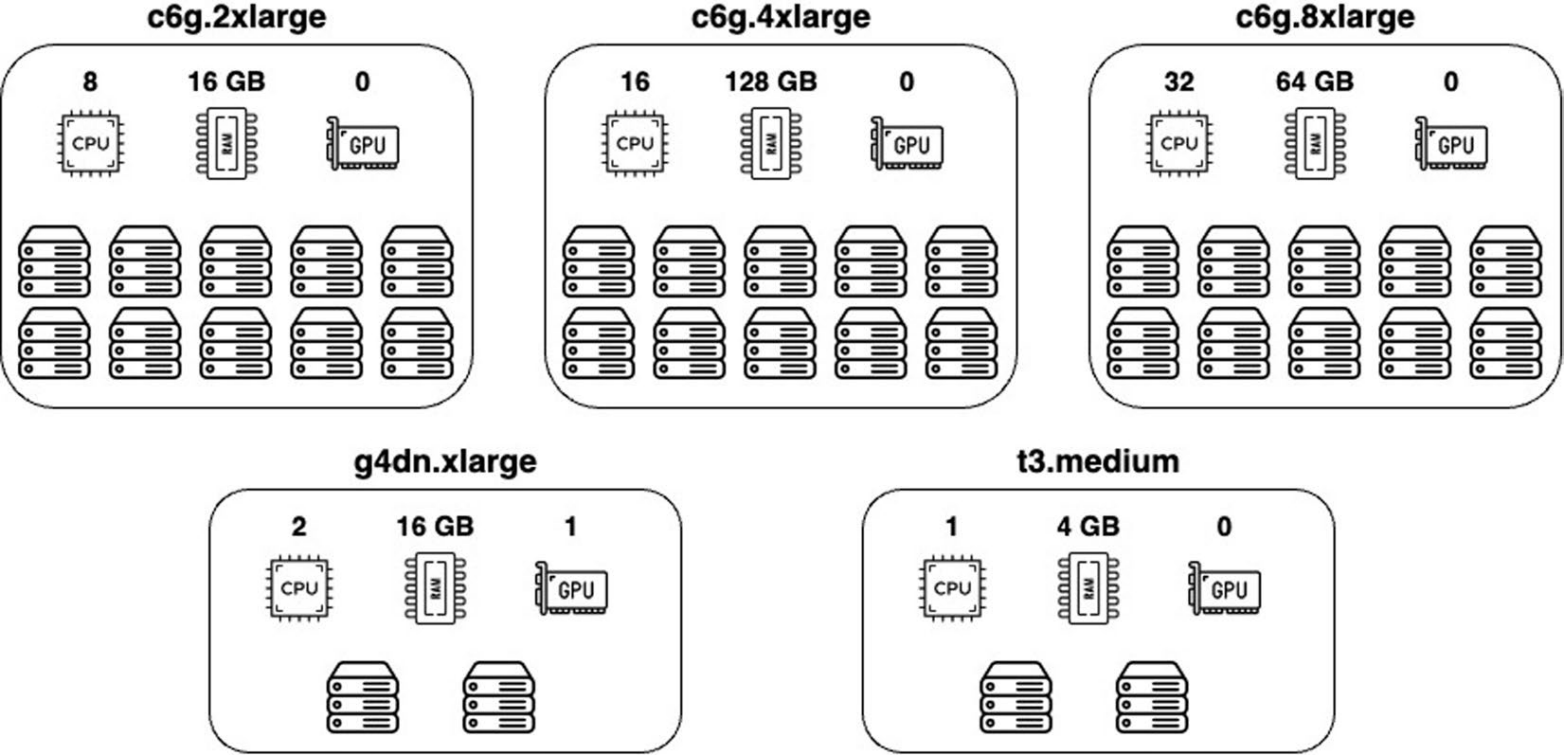
AWS infrastructure specifications.

Herein, an heterogeneous DC *S* offers the set of resources *R* = {CPU cores, memory, GPU}, and is comprised of 34 nodes with 572 CPU cores, 2120 GB of memory and 2 GPU units in total. Each node is characterized by a type (e.g., medium, xlarge, 2xlarge, 4xlarge, and 8xlarge) which determines its maximum number of CPU threads, its memory capacity, and its GPU units. Figure 3 provides an overview of the nodes comprising our AWS testbed, along with their specifications.

### Workload Scenarios

**Fig. 4:**
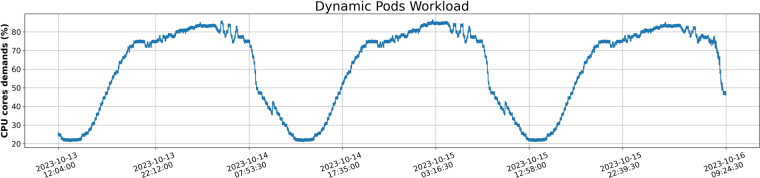
Dynamic Workload of pods across time depicting the CPU demands of applications in percentage.
- **Scenario B - Static**: This scenario is comprised of resource demands that are characterized by the stability of fixed patterns, which, as their name implies, do not exhibit any load volatility. The majority of resource demands in this scenario originate from the 420 pods of the base-fixed deployment, with the contribution of the remaining deployments being limited to the overall resource demand. Figure 5 depicts a Static workload, where it seems that the CPU demands are steadily around 84% during the date.

For the workload cases, three distinct scenarios were designed to encompass all occasions within the telco data centers as described in Table 1. These scenarios are characterized by their varying proportions of pods, each adhering to different workload patterns – diurnal, staggered, or fixed. In the dynamic scenario, for example, the workload consists of pods exhibiting dynamic fluctuations, with a predominant emphasis on those following the diurnal pattern. Following, the different scenarios are explained:

- **Scenario A - Dynamic**: In this case, pods of diurnal deployments yield the largest fraction of resource demand compared to staggered and fixed patterns, indicating an anticipation of high variations in overall resource demands. Figure 4 represents a dynamic workload during the days with high demands during the daytime ( ≈ 80%) and decreased demands around the night ( ≈ 20%).

**Fig. 5:**
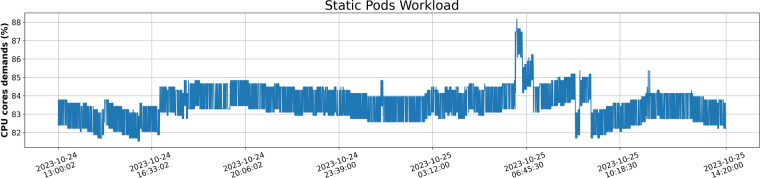
Static Workload of pods across time depicting the CPU demands of applications in percentage.
- **Scenario C - Balanced**: In the balanced scenario, which involves a harmonious distribution of resources, there are 284 pods of the base-fixed deployment and 70 pods of the base-diurnal deployment. This combination results in a well-distributed and moderate load across the deployments, contributing to a balanced resource demand profile. Figure 6 shows a Balanced workload with augmented needs in resources at day ( ≈ 85%) and less, but still high, requirements around the off-peak period ( ≈ 60%).

**Fig. 6:**
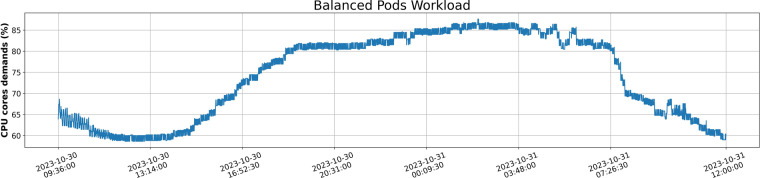
Balanced Workload of pods across time depicting the CPU demands of applications in percentage.

## Usage Notes

The proposed dataset serves as a critical contribution to the telco research domain given the current shortage of openly available data related to telco workload resource demands. In the following, we elaborate on three use cases which can be facilitated by the proposed dataset.

### Strategic Network Configuration

In the dynamic landscape of telecommunications operations, the telco workload dataset emerges as a functional tool. This resource provides telco operators with the means to strategically configure their networks, going beyond the basics of optimizing resource management and server allocation. Notably, the dataset’s richness in diverse resource types, such as CPU cores and Memory, empowers operators to examine cross-resource correlations, gaining useful insights. This depth of understanding is pivotal for developing sophisticated prediction models, enabling operators to anticipate spikes in demand and proactively adjust network configurations. Additionally, the dataset proves invaluable for proactive decision-making, allowing operators to spawn additional load balancers or defer maintenance/upgrade tasks in anticipation of workload increase. In essence, the versatility of the dataset stands as a foundation for telco operators, amplifying their capacity to make informed decisions across a spectrum of network-related scenarios. This, in turn, elevates the overall efficiency and resilience of telecom infrastructures.

### Data Center Sizing

In the realm of infrastructure management, the persistent challenge of underutilized servers during off-peak periods leads to inefficient resource allocation and unnecessary energy consumption within data centers. Leveraging these comprehensive workloads, providers can devise robust methodologies to dynamically determine the required number of servers over time. A viable solution involves workload consolidation–transferring tasks from underutilized servers to those with higher usage and subsequently shutting down the former. This decision-making ability significantly enhances resource management, ensuring computational resources are allocated precisely when needed, thereby optimizing energy usage and overall operational costs. This adaptive server utilization approach minimizes energy waste during low-demand periods while aligning with the demand curve to maintain resource availability.

### Proactive Data Center Sizing

**Fig. 7:**
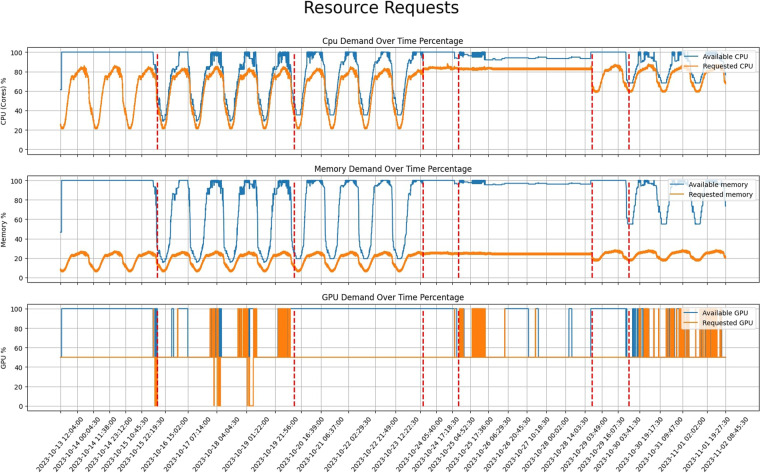
A visual representation of the three resource types (CPU, Memory, GPU) over time. With orange is depicted the aggregated requested demands of pods in percentage and with blue is the available resources that the infrastructure provides to accommodate the applications.

The dataset’s usability extends to a combined application of two steps: forecasting the future resource demands of the data center^16^ and, based on predictions, determining the necessary nodes to efficiently serve the load. This integrated approach allows for the activation of only the essential servers, with the remaining being deactivated to minimize energy consumption and reduce operational expenses. In the conducted experiments within the AWS infrastructure detailed in the Infrastructure Setup section, the provided workloads were employed to train machine learning algorithms for forecasting resource demands. Subsequently, decision-making algorithms were implemented to ascertain the optimal number of nodes required in the forthcoming hours, aligning with anticipated pod demands. Figure 7 visually represents the requested and available resources (CPU, memory, GPU) in the infrastructure across time, separated by different experiments outlined in Table 3. The available resources were determined through the decision-making process, where the optimal number of servers was selected. Additionally, each scenario included a fallback strategy; if the algorithms deactivated more servers and subsequently encountered pods that couldn’t be accommodated, some servers would be reactivated. Notably, during the Data Collection phase, no algorithms operated, and all servers remained active. Observing the results and Fig. 7, scenario 2 emerged as the most notable experiment, exhibiting the highest gain by utilizing only the necessary number of servers. Detailed information on node availability is stored in the Zenodo repository^15^ in the file named *nodes_allocatable*, also available on Github. The file is devoid of any missing values, except for the *Status* and *Condition* columns, where NaN values indicate that the node is inactive at that timestamp.

**Table 3:** Experiments description.

Experiment	Time Period	Type	Gain	Overprediction	Description / Comments
1	3 days	Data Collection	—	—	Scenario A - Dynamic Resource Demands
2	4 days	Algorithms Execution	31%	0%	This is the case where the higher cost saving was achieved. It didn’t use overprediction, which means that only essential nodes were active, but with cases of long pending pods that couldn’t be served
3	4 days	Algorithms Execution	26%	10%	The second better case based on gain. In this scenario, both increased gain and decreased long pending pods were achieved.
4	1 day	Data Collection	—	—	Scenario B - Static Resource Demands
5	4 days	Algorithms Execution	6%	10%	This is the less energy and cost saving case where the demand is fixed and almost all the nodes are up and running. In general, in a static environment, it is difficult to save too much.
6	1 day	Data Collection	—	—	Scenario C - Balanced Resource Demands
7	3 days	Algorithms Execution	14%	10%	The average case with the balanced workload. Herein, a moderate gain is achieved.

## Data Availability

The code structure demonstration is available in the README file in the GitHub repository: https://github.com/athenarc/arcadia-project/tree/master/resource-demand-open-data. The code demonstration contains two folders: data, and notebook. The data directory shows all the original data with the resource demands per scenario and the *nodes_allocatable* file with records from the experiments analyzed in the section *Usage Notes -> Proactive Data Center Sizing* and the notebook directory includes a notebook containing the visualizations of data. Python 3 is used for data processing, analysis, visualization, and setup.
